# Genetic risk for severe COVID‐19 correlates with lower inflammatory marker levels in a SARS‐CoV‐2‐negative cohort

**DOI:** 10.1002/cti2.1292

**Published:** 2021-06-06

**Authors:** Timothy R Powell, Matthew Hotopf, Stephani L Hatch, Gerome Breen, Rodrigo RR Duarte, Douglas F Nixon

**Affiliations:** ^1^ Division of Infectious Diseases Department of Medicine Weill Cornell Medicine New York NY USA; ^2^ Social, Genetic & Developmental Psychiatry Centre Institute of Psychiatry, Psychology & Neuroscience King's College London London UK; ^3^ Department of Psychological Medicine Institute of Psychiatry, Psychology & Neuroscience King's College London London UK; ^4^ South London and Maudsley NHS Foundation Trust London UK

**Keywords:** COVID‐19, genetic risk, IFN‐γ, inflammation, SARS‐CoV‐2, TNF‐α

## Abstract

**Objectives:**

It remains unknown how inflammatory marker levels differ amongst individuals susceptible to coronavirus disease 2019 (COVID‐19), prior to severe acute respiratory syndrome coronavirus 2 (SARS‐CoV‐2) infection and the onset of the cytokine storm. We used genetic risk scores to model how susceptibility to severe COVID‐19 correlates with baseline levels of 35 inflammatory markers, by testing their impact in a SARS‐CoV‐2‐negative population cohort. Because of the established effects of age and body mass index on severe COVID‐19 risk, we further considered how these variables interacted with genetic risk to affect inflammatory marker levels.

**Methods:**

We accessed data on 406 SARS‐CoV‐2‐negative individuals as part of a UK population study. Multiplex electrochemiluminescence methods were applied to blood serum, and 35 inflammatory markers were assayed. Corresponding genotype data, alongside results from a large genome‐wide association study of severe COVID‐19, allowed us to construct genetic risk scores and to test their impact on inflammatory protein levels.

**Results:**

Our results revealed that a higher genetic risk for severe COVID‐19 was associated with lower blood levels of interferon gamma (IFN‐γ), vascular endothelial growth factor D (VEGF‐D) and tumor necrosis factor alpha (TNF‐α). Inflammatory profiles of those with high genetic risk increasingly diverge from the norm in association with age and obesity.

**Conclusion:**

Our results support the theory that individuals at risk of severe COVID‐19 have a deficient innate immunity marked by reduced levels of inflammatory markers at baseline, including IFN‐γ, VEGF‐D and TNF‐α. We hypothesise that a secondary overactive adaptive immune response may subsequently explain the high levels of cytokines observed in SARS‐CoV‐2‐positive COVID‐19 patients.

## Graphical abstract

We used genetic risk scores to model how susceptibility to severe COVID‐19 correlates with baseline levels of 35 inflammatory markers, by testing their impact in a SARS‐CoV‐2‐negative population cohort. Higher genetic risk for severe COVID‐19 was associated with lower blood levels of interferon gamma (IFN‐γ), vascular endothelial growth factor D (VEGF‐D) and tumor necrosis factor alpha (TNF‐α). Inflammatory profiles of those with high genetic risk increasingly diverge from the norm in association with age and obesity.

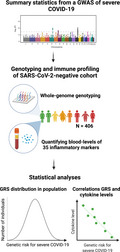

## Introduction

There is a considerable degree of interindividual variability in response to severe acute respiratory syndrome coronavirus 2 (SARS‐CoV‐2) infection, with host genetics, demographic and environmental factors playing key roles in moderating prognosis.^1^, ^2^ Whilst some individuals remain symptom‐free upon infection, others develop coronavirus disease 2019 (COVID‐19) symptoms including fever, cough and breathing difficulties, and some ultimately die.^3^

The sudden and rapid release of inflammatory proteins, commonly referred to as the ‘cytokine storm’, represents one pathological mechanism proposed to contribute to COVID‐19.^4^ This theory has largely gained gravitas because of case–control studies which have consistently revealed heightened levels of proinflammatory markers in the blood of moderate and severe COVID‐19 patients relative to uninfected, healthy participants.^5^ For instance, interleukin 6 (IL‐6), tumor necrosis factor alpha (TNF‐α) and interferon gamma (IFN‐γ) show heightened circulating levels in cases, and rapid rises alongside COVID‐19 progression and severity, which suggest they may represent promising indicators of disease prognosis.^6^, ^7^, ^8^

Whilst these findings represent an encouraging advance in the field, important questions remain as to why some individuals show such drastic increases in inflammatory markers, whilst others do not, and whether or not levels of inflammatory cytokines prior to infection, moderate susceptibility to COVID‐19. Higher levels of inflammatory proteins at baseline amongst susceptible individuals might support the applicability of targeted anti‐inflammatories as a prophylaxis or imply that higher levels of cytokines observed amongst acutely infected patients are inflated because of the presence of pre‐existing baseline differences. Alternatively, if lower levels of cytokines are observed at baseline amongst susceptible individuals, it may suggest a compromised innate immune system and consequently an increased chance of infection. Regardless of the direction of effect, the exploration of interindividual baseline differences in inflammatory marker levels could allow for biomarker development capable of differentiating individuals who are susceptible to severe COVID‐19 prior to infection, potentially improving patient management or identifying those most in need of safeguarding. Currently, however, there has been a marked absence of longitudinal studies that encompass time points preceding infection, and thus, we do not currently know how baseline inflammatory profiles differ amongst individuals who are susceptible to severe COVID‐19.

**Figure 1:**
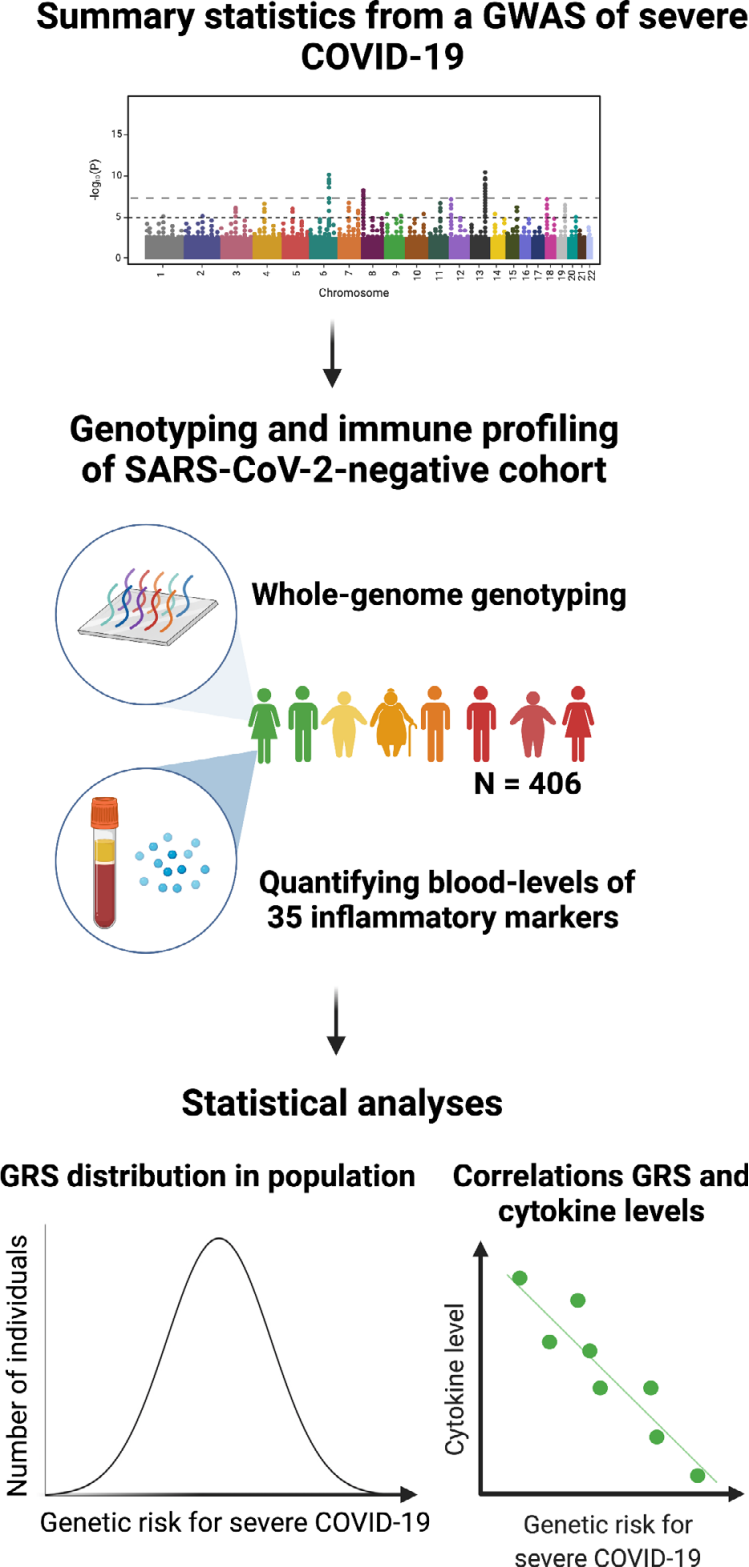
An overview of our approach.

Here, we modelled how susceptibility to severe COVID‐19 expresses itself in the immune system prior to SARS‐CoV‐2 infection. To achieve this, we utilised data from the largest genome‐wide association study (GWAS) of severe COVID‐19 to date ^1^ and derived genetic risk scores (GRS) that encapsulate the cumulative effect thousands of genetic variants exert on susceptibility. These quantitative genetic scores can be used to establish those at highest or lowest genetic risk in independent populations, where corresponding genotype data are available. We studied how GRS for severe COVID‐19 affected blood levels of 35 inflammatory markers in a SARS‐CoV‐2‐negative population cohort comprising of over 400 individuals from London, UK (Figure 1). Our primary aim was to test the effect genetic risk exerts on baseline levels of inflammatory markers prior to SARS‐CoV‐2 infection and the onset of the cytokine storm. As a secondary aim, we considered how genetic risk interacted with age and body mass index (BMI) to affect inflammatory marker levels. We investigated this because the risk of severe COVID‐19 and associated mortality is higher amongst elderly and obese individuals, and because we have previously demonstrated a substantial influence of age and BMI on inflammatory marker levels.^9^, ^10^, ^11^, ^12^ Our results shed important new insights into possible immunological mechanisms moderating risk for severe COVID‐19.

## Results

### Genetic susceptibility to severe COVID‐19 is associated with lower levels of TNF‐α, VEGF‐D and IFN‐γ in a SARS‐CoV‐2‐negative population cohort

**Figure 2:**
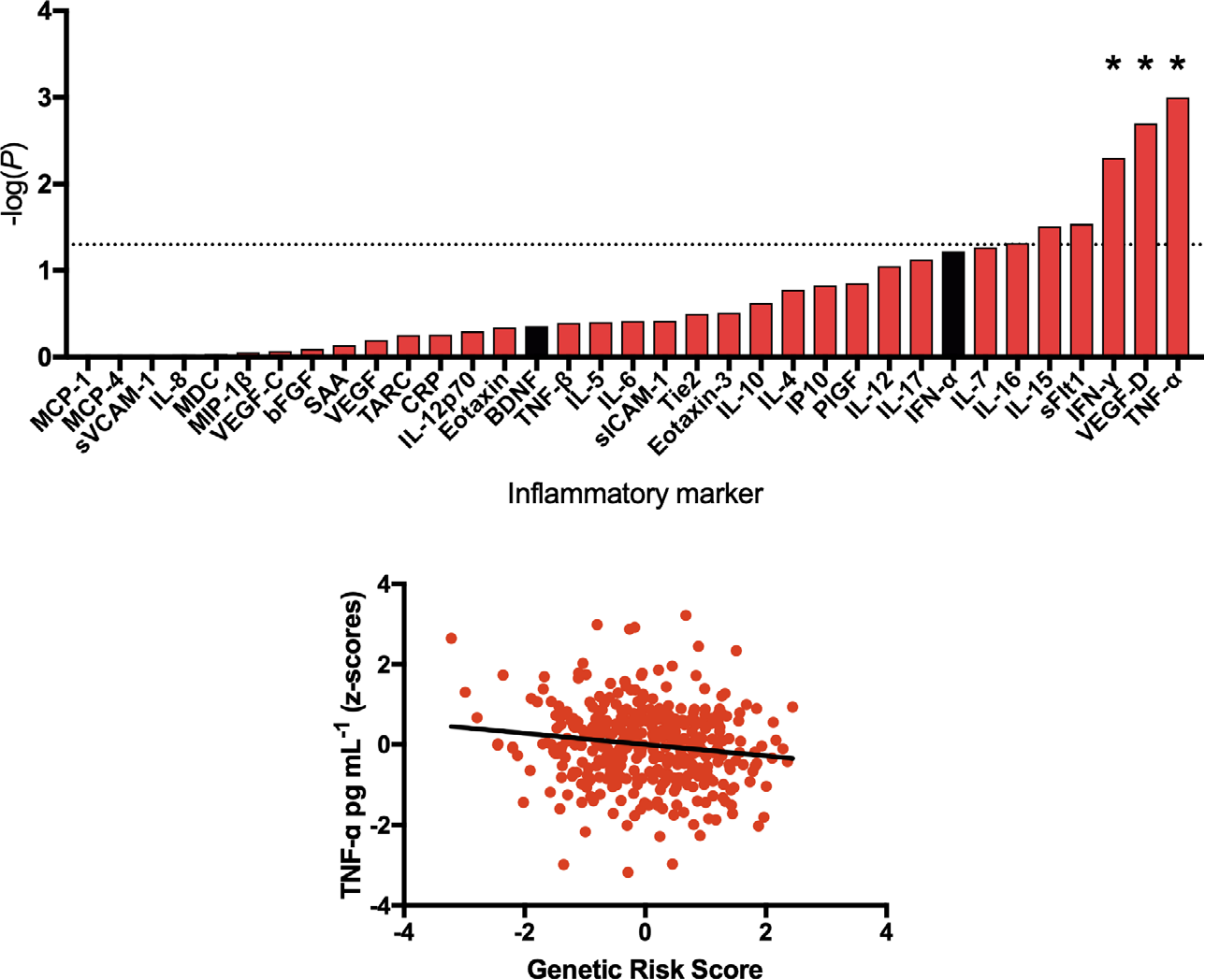
The association between genetic risk for severe coronavirus disease 2019 and inflammatory marker levels in a severe acute respiratory syndrome coronavirus 2‐negative cohort. Top: Results from linear regressions displayed as a bar plot. The strength of association between genetic risk scores and inflammatory marker levels is indicated by −log_10_(*P*), whereby taller bars represent higher levels of significance. Red bars correspond to a negative association (β) between the genetic risk score and inflammatory marker level, whereas black bars correspond to a positive association. The dashed line represents the nominal significance threshold (*P* < 0.05), *represents *P*_FDR_ < 0.1. Bottom: A scatterplot showing the relationship between genetic risk (corrected for seven population covariates) and TNF‐α levels (corrected for cytokine assay batch, age, gender, body mass index, ethnicity and smoking status).

Linear regressions revealed a negative relationship between genetic risk for severe COVID‐19 and levels of TNF‐α [β = −0.065 (−0.105, −0.025), *P* = 0.001, *P*_FDR_ = 0.035, *R* ^2^ = 0.009], vascular endothelial growth factor D [VEGF‐D; β = −0.072 (−0.118, −0.026), *P* = 0.002, *P*_FDR_ = 0.035, *R* ^2^ = 0.017] and IFN‐γ [β = −0.137 (−0.232, −0.042), *P* = 0.005, *P*_FDR_ = 0.058, *R* ^2^ = 0.018](Figure 2). This negative relationship was mirrored in the majority of inflammatory markers as marked in red, Figure 2 (top), and the most significant association is depicted in Figure 2 (bottom).

### Age interacts with genetic susceptibility for severe COVID‐19 to affect inflammatory marker levels in a SARS‐CoV‐2‐negative population cohort

**Figure 3:**
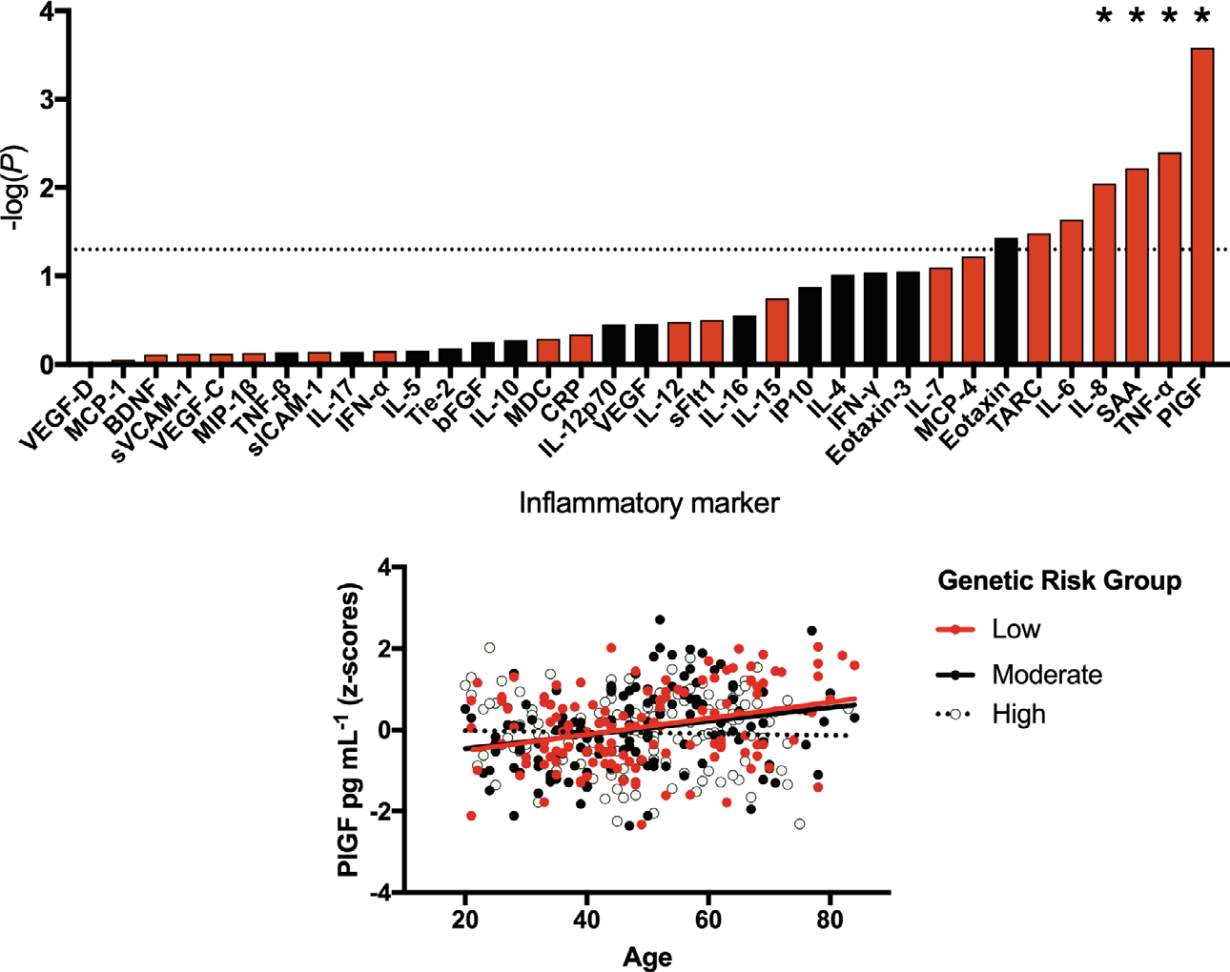
The interaction between genetic risk for severe coronavirus disease 2019 (COVID‐19) and age on inflammatory marker levels in a severe acute respiratory syndrome coronavirus 2‐negative cohort. Top: Results from linear regressions displayed as a bar plot. The interaction between genetic risk for severe COVID‐19 and age was tested in relation to inflammatory marker levels, with the strength of association represented by −log_10_(*P*), whereby taller bars represent higher levels of significance. Red bars correspond to a negative association (β), whereby older individuals with higher genetic risk have the lowest levels of an inflammatory marker, whereas black bars correspond to a positive association in which older individuals with higher genetic risk have higher levels of an inflammatory marker. The dashed line represents the nominal significance threshold (*P* < 0.05), *represents *P*_FDR_ < 0.1. Bottom: A scatterplot demonstrating the interaction between age and genetic risk on blood levels of PIGF (corrected for cytokine assay batch, gender, body mass index, ethnicity and smoking status).

Given the higher risk of severe COVID‐19 amongst older individuals,^12^ we tested whether age modified the relationship between genetic risk and inflammatory marker levels. We found significant interactions between age and genetic risk for severe COVID‐19 on levels of placental growth factor, PlGF [β = −0.020 (−0.031, −0.009), *P* = 0.0003, *P*_FDR_ = 0.009, *R* ^2^ = 0.022], TNF‐α [β = −0.014 (−0.024, −0.004), *P* = 0.004, *P*_FDR_ = 0.070, *R* ^2^ = 0.007], SAA [β = −0.052 (−0.089, −0.015), *P* = 0.006, *P*_FDR_ = 0.070, *R* ^2^ = 0.014] and IL‐8 [β = −0.022 (−0.039, −0.006), *P* = 0.009, *P*_FDR_ = 0.071, *R* ^2^ = 0.017] (Figure 3 top).

**Figure 4:**
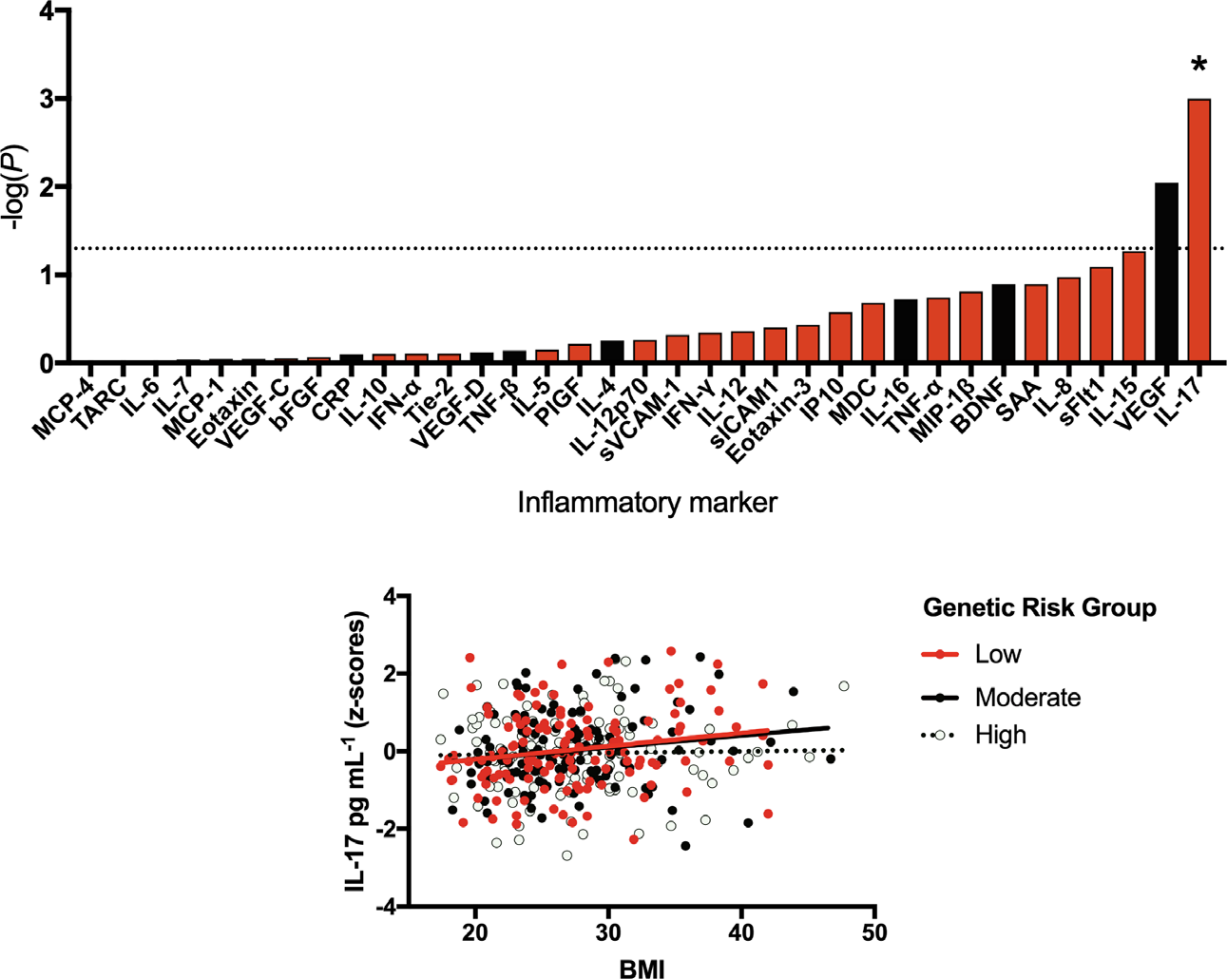
The interaction between genetic risk for severe coronavirus disease 2019 (COVID‐19) and body mass index (BMI) on inflammatory marker levels in a severe acute respiratory syndrome coronavirus 2‐negative cohort. Top: Results from linear regressions displayed as a bar plot. The interaction between genetic risk for severe COVID‐19 and BMI was tested in relation to inflammatory marker levels, with the strength of association represented by −log_10_(*P*). Red bars correspond to a negative association (β), whereby individuals with a higher genetic risk and higher BMI have the lowest levels of an inflammatory marker, whereas black bars correspond to a positive association in which individuals with a higher genetic risk and a higher BMI have higher levels of an inflammatory marker. The dashed line represents the nominal significance threshold (*P* < 0.05), *represents *P*_FDR_ < 0.1. Bottom: A scatterplot demonstrating the interaction between BMI and genetic risk on blood levels of IL‐17 (corrected for cytokine assay batch, gender, age, ethnicity and smoking status).

Continuous GRS were used in all of our analyses (including interactions), but to aid the interpretability of our interaction plots (Figure 3, bottom; Figure 4, bottom), we converted our quantitative measure of genetic susceptibility to severe COVID‐19 into an ordinal variable, separating individuals into three equally sized groups corresponding to low, moderate and high genetic risk groups. We observed that this grouping explained 81% of the variance in relation to our quantitative genetic risk measure for severe COVID–19 in the South East London Community Health Study (SELCoH) cohort, corrected for seven population covariates [β = 1.089 (1.037, 1.141), *P* = 1.66 × 10^−146^], thus representing an adequate proxy. As demonstrated in Figure 3 (bottom), whilst low and moderate genetic risk groups exhibited higher levels of PlGF in association with age, there was no positive association between age and PlGF observed in the high genetic risk group. This trend was similar for all four inflammatory markers significantly affected by genetic risk by age interactions.

### Body mass index interacts with genetic risk for severe COVID‐19 to affect IL‐17 levels in a SARS‐CoV‐2‐negative population cohort

Given that obesity is a risk factor for severe COVID‐19,^12^ we tested whether genetic risk interacted with BMI to affect inflammatory marker levels. We found that genetic risk for severe COVID‐19 interacted with BMI to significantly affect levels of IL‐17 [β = −0.046 (−0.073, −0.02), *P* = 0.001, *P*_FDR_ = 0.035, *R* ^2^ = 0.027] (Figure 4 top). Whilst those with low‐to‐moderate genetic risk demonstrated higher levels of IL‐17 in association with BMI, there was no relationship observed in the high genetic risk group (Figure 4 bottom).

### Sensitivity analyses

Because of the absence of a predefined *P*‐value threshold (and SNP combination) that best captures genetic risk for COVID‐19 in independent cohorts, we selected an arbitrary *P*‐value threshold (*P*_T_) of 0.5. This was selected based on earlier studies which revealed that polygenic phenotypes in moderately powered GWAS are better captured using a relaxed *P*‐value cut‐off, as used in our prior work in relation to genetic risk for HIV‐1 acquisition and inflammatory marker levels.^13^ To explore the reliability of the findings identified above and to ensure results were not an artefact related to this single arbitrary *P*_T_, we generated GRS for severe COVID‐19 using a wider range of *P*_T_: 0.001, 0.05, 0.1 and 0.3. We then repeated the regressions described above. These analyses confirmed that GRS generated using different *P*‐value thresholds produced similar results, and we identified which threshold explained the most variance in inflammatory marker levels, Table 1. To confirm that our main effect results (optimal *P*_T_ = 0.3) were not being confounded by our mixed population, we repeated this regression in a White only subset. We confirmed that 98% of the self‐reported White individuals (283/290) in SELCoH were of European ancestry. We achieved this by merging SELCoH genotype data with a 1000 Genomes reference panel. Principal components 1 and 2 were then generated, and all SELCoH samples whose Euclidean distance fell within a defined radius of known Europeans (europeanTh, scaling factor = 1) were confirmed as White.^14^ When repeating our main effects analysis in this smaller sample subset, we confirmed a consistent direction of effect in relation to TNF‐α [β = −0.017 (−0.028, −0.006), *P* = 0.003], VEGF‐D [β = −0.102 (−0.204, 3.0 × 10^−4^), *P* = 0.051] and IFN‐γ [β = −0.100 (−0.214, 0.014), *P* = 0.086].

**Table 1:** Results from our sensitivity analyses confirm that genetic risk scores (GRS) generated using alternative *P*‐value thresholds also significantly predict levels of inflammatory markers

Test	Inflammatory marker	Significant at multiple *P*_T_?	*P*_T_ explaining most variance	β (95% CI)	*P*‐value	*R* ^2^
GRS	TNF‐α	Y	0.3	−0.071 (−0.111, −0.031)	0.001	0.010
	VEGF‐D	Y	0.3	−0.076 (−0.122, −0.029)	0.001	0.018
	IFN‐γ	Y	0.3	−0.148 (−0.244, −0.052)	0.003	0.021
GRS*Age	PlGF	Y	0.3	−0.020 (−0.031, −0.009)	2.270 x 10^−4^	0.022
	TNF‐α	Y	0.3	−0.015 (−0.025, −0.005)	0.002	0.008
	SAA	Y	0.3	−0.051 (−0.088, −0.014)	0.007	0.013
	IL‐8	Y	0.3	−0.023 (−0.039, −0.006)	0.007	0.016
GRS*BMI	IL‐17	Y	0.001	−0.046 (−0.071, −0.021)	3.660 x 10^−4^	0.030

## Discussion

Patients with severe COVID‐19 demonstrate heightened circulating levels of inflammatory cytokines which are hypothesised to contribute to disease pathology; however, little is understood about whether or not a heightened proinflammatory state is also present at baseline (prior to infection), or whether compromised levels of inflammation increase risk for a systemic SARS‐CoV‐2 infection and severe COVID‐19.^15^ Therefore, this study sought to understand the effect risk for severe COVID‐19 imparts on the immune system prior to infection and the onset of the cytokine storm. To achieve this, we studied the effect genetic risk exerts on levels of 35 inflammatory markers in the blood of a SARS‐CoV‐2‐negative population cohort. The derivation and study of risk in a population cohort has a number of key advantages when trying to isolate etiological risk mechanisms, including the reduction of clinical and demographic confounders commonly present in cases.^16^ Overall, the results from our work have a number of potentially important implications.

First, our study suggests that genetic risk for severe COVID‐19 is associated with broadly lower levels of inflammatory proteins and particularly IFN‐γ, TNF‐α and VEGF‐D, Figure 2. This finding supports hypotheses that suggest lower levels of innate immunity (e.g. antiviral activity) predispose particular individuals to SARS‐CoV‐2 infection and COVID‐19, and complements epidemiological research which has shown that vaccines (e.g. influenza and tuberculosis), known to broadly prime the immune system, may be partially protective.^17^, ^18^, ^19^

Second, our results suggest caution is needed when interpreting case–control data and particularly when using it to rank the relative importance of inflammatory markers in relation to COVID‐19, as the baseline differences identified here mean we are probably underestimating the importance of some cytokines. For instance, a COVID‐19 case–control study found that IFN‐γ and TNF‐α show more modest relative differences than IL‐6 in patients.^20^ However, our data suggest that baseline levels of IFN‐γ and TNF‐α are lower to begin with amongst susceptible individuals. Therefore, it is plausible that we are underestimating the importance of these two cytokines and​, if assessed longitudinally, the absolute changes observed following infection might be greater than the differences expected based on case‐control data. This could explain why high levels of IL‐6 in mouse, and *in vitro*, do not appear to mimic COVID‐19 pathology, whereas treatment with combined IFN‐γ and TNF‐α does. Indeed, recent single‐cell RNA sequencing and functional data suggest that a synergistic elevation of IFN‐γ and TNF‐α recapitulates the pathological and biochemical effects of COVID‐19 observed both in the immune system and in lungs of severe patients.^21^ Furthermore, the combined inhibition of IFN‐γ and TNF‐α during an acute infection reduced the mortality associated with SARS‐CoV‐2 infection in a mouse model. Thus, our results extend this hypothesis by indicating that a relatively deprived TNF‐α/IFN‐γ environment at baseline, in conjunction with a sudden rise, as observed in patients and *in vitro*,^21^ could partially explain the conversion of a SARS‐CoV‐2 infection to severe COVID‐19.

Third, we explored whether two established risk factors for severe COVID‐19, age and BMI,^12^ interact with genetic risk to affect inflammatory marker levels. In relation to age, we found that levels of four inflammatory markers were significantly predicted by our interaction term, whereby older individuals with higher genetic risk had lower levels of PlGF, TNF‐α, SAA, IL‐8, relative to their peers with lower genetic risk. Our previous research in a cohort of over 1000 participants had demonstrated positive associations between age and all of these inflammatory marker levels,^10^ suggesting this correlation likely represents a normal part of ageing, or ‘inflammaging’.^22^ However, amongst those with high genetic risk for severe COVID‐19, we fail to see an age‐associated rise, Figure 3. This may suggest that for these individuals, their inflammatory profile (and potentially innate immune system) is relatively stable and fixed. Whilst reduced levels of inflammaging may be beneficial in reducing risk of some age‐related inflammatory diseases, it may result in ageing immune cells offering less protection against pathogens.^22^ Similarly, we observe the same flat relationship between BMI and IL‐17 levels in those with high genetic risk for severe COVID‐19, Figure 4, suggesting the innate immune system of these individuals may be less plastic across the life course than those with a low‐to‐moderate genetic risk.

Despite the promising results described here, our study has a number of limitations which should be acknowledged. First, the effect sizes observed are small and further work will be needed to understand whether these statistically significant effects are biologically or clinically meaningful. Our power to detect effects is also limited by the size of the current GWAS for severe COVID‐19 and our relatively small target dataset. Future larger samples will allow for more powerful inferences. Second, whilst it is tempting to think about these inflammatory markers as playing causal roles, we can only infer *associations,* as GRS may encompass SNPs related to other independent mechanisms affecting cytokine levels (i.e. via horizontal pleiotropy). Future well‐powered GWAS may allow for Mendelian randomisation studies where we can better discern cause from effect. However, it should be noted that a transcriptome‐wide association study (TWAS) performed on the same COVID‐19 GWAS data we utilise here, revealed a direct effect of risk SNPs on the downregulated expression of TNF‐ and IFN‐related genes, and thus, there is at least some support for a direct causal role for these two cytokines.^1^ In relation to interferon signalling, the TWAS revealed that interferon alpha and beta receptor subunit 2 (*IFNAR2*) transcripts are downregulated in association with genetic risk for severe COVID‐19, which could partially explain our results pertaining to IFN‐γ. For instance, *Ifnar2* knockout mice demonstrate lower levels of IFN‐γ during early influenza infection and increased levels during later infection,^23^ which parallels our theory of a lowered initial inflammatory response to SARS‐CoV‐2, followed by an exaggerated one, in those genetically susceptible to severe COVID‐19. Finally, we used GRS applied to a SARS‐CoV‐2‐negative population, because of the inherent difficulties of accessing data from longitudinal COVID‐19 cytokine studies which include a SARS‐CoV‐2‐negative baseline. Future large, prospective longitudinal studies will be best placed to confirm the inferences made here and to refine our understanding of the temporal changes to cytokines before and after infection.

To conclude, our results support the theory that individuals at risk of severe COVID‐19 have a deficient innate immunity marked by reduced levels of circulating inflammatory proteins such as IFN‐γ and TNF‐α. In light of the previously reported cytokine storm found post‐infection, we surmise that a secondary overactive adaptive immune response may subsequently explain the high levels observed in severe COVID‐19 patients, as the immune system attempts to compensate for an insufficient first‐line response.

## Methods

### The South East London Community Health Study

The SELCoH study is a probability sampled general population survey designed to investigate mental and physical health in the general population in London, UK.^24^ The first phase aimed to assess common physical and mental disorders in South East London; the second, to examine the roles of historical social context and policy in shaping patterns of health inequalities; and the third, to collect biological specimens from a subset of participants, including blood for serum separation and DNA for genotyping.

We have previously used genetic, demographic, clinical and inflammatory marker data within SELCoH to investigate a broad range of questions, including the effects genetic risk for HIV‐1 acquisition, BMI and major depressive disorder exert on inflammatory cytokine levels.^9^, ^13^ Here, we applied a similar strategy in order to test how genetic risk for severe COVID‐19 correlates with levels of 35 inflammatory proteins. As the collection of this sample substantially preceded the first diagnosis of COVID‐19, all participants are assumed to be SARS‐CoV‐2‐negative at the time blood was drawn.

The subsample analysed here consisted of 406 individuals for which both inflammatory and genetic data were available, as described previously.^11^, ^13^ The mean age of our sample was 48.7 ± 15.1 (standard deviation), with a mean BMI of 27.3 ± 5.5. The cohort is representative of the source population and consisted of 45.3% males; 20.9% current smokers and 40.4% ex‐smokers; and 56.8% White British, 14.6% White Other, 10.9% Black African, 8.4% Black Caribbean, 6.2% non‐White other and 3.2% Mixed. Participants received detailed and repeated phenotypic assessments as part of three separate phases of SELCoH.

### Ethics statement

The SELCoH study received approval from King's College London Research Ethics Committee, reference PNM/12/13‐152. Informed written consent was obtained from all participants at the time of sample collection.

### Data availability statement

Because of ethical restrictions, SELCoH data are not publicly available. Access to SELCoH data requires local approval (contact: selcoh@kcl.ac.uk).

### Quantification and analysis of cytokines

Serum levels (pg mL^−1^) of 35 blood‐based markers were assessed in blood samples from the SELCoH cohort using multiplex ELISA‐based technology provided by the Meso Scale Discovery Biomarker kits, as described previously.^9^, ^10^, ^11^, ^13^

### DNA genotyping

DNA samples from the SELCoH cohort were sent to the Affymetrix Research Services Laboratory in Santa Clara, CA, USA. Genotyping was assayed using the UK Biobank Axiom Array (r3) which comprises of 820 967 genetic markers (Affymetrix, Santa Clara, CA, USA). Genotype imputation was performed on the Michigan Imputation Server v1.2.4 (https://imputationserver.sph.umich.edu/index.html), using Eagle v2.4 phasing and the 1000 Genomes Phase 3 v5 (mixed population) as a reference panel. Genotype data underwent standard quality control procedures as described previously.^9^, ^13^

### GWAS summary statistics

We obtained GWAS summary statistics from the largest study to date comparing genetic differences in severe UK COVID‐19 patients with life‐threatening symptoms versus matched controls.^1^ Data were available for public download via the Genetics Of Mortality In Critical Care website (https://genomicc.org/data/), where GWAS summary statistics were stratified by ethnicity. Due to the higher number of Europeans in this COVID‐19 GWAS (and hence greater power) and the fact that the majority of the SELCoH sample consisted of White Europeans (> 70%), we utilised data pertaining to European ancestry cases (*n* = 1676) versus matched controls (*n* = 8380).

### Genetic risk scores

Individualised GRS within the SELCoH sample were calculated using PRSice‐2, a GRS quantification software.^25^ This pipeline uses summary statistics from a base GWAS (e.g. COVID‐19) to generate individualised risk scores in a target dataset (e.g. SELCoH). Briefly, the number of risk alleles in the base dataset is multiplied by SNPs’ effect sizes to generate individualised GRS in the target dataset. PRSice‐2 clumps SNPs in the genotype files of the target dataset and removes those in high linkage disequilibrium to avoid inflation. For our initial screen, we output a GRS using all SNPs in the GWAS with a *P*‐value threshold (*P*_T_) < 0.5. This arbitrary threshold was selected based on earlier studies which revealed that polygenic phenotypes in moderately powered GWAS are better captured using a relatively relaxed *P*‐value cut‐off, and as used in our prior work in relation to genetic risk for HIV‐1 acquisition and inflammatory marker levels.^13^ For our sensitivity analyses, we output GRS using *P*_T_ = 0.001, 0.05, 0.1 and 0.3, where we confirmed associations observed at *P*_T_ = 0.5. This practice is relatively standard when the optimal combination of SNPs (i.e. those under a specific *P*‐value threshold in the base GWAS) has yet to be determined in independent cohorts of patients, and is used to add support that results obtained from an arbitrary *P*_T_ are not a spurious effect related to that single threshold.

As a second sensitivity test, we validated that our significant main effects were consistent in the White European subset of our sample (71.4% of the total sample, based on self‐report). This was to double‐check that our analyses were not being confounded by our mixed ancestry population. To achieve this, we merged SELCoH genotype data with a 1000 Genomes reference panel. Principal components 1 and 2 were then generated, and all SELCoH samples whose Euclidean distance fell within a defined radius of known Europeans (europeanTh, scaling factor = 1) were confirmed as White, using plinkQC.^14^

### Statistical analysis

For both main effects and interaction analyses, we included the following as covariates: age, gender, BMI, self‐reported ethnicity, smoking status (never/prior/current), seven population covariates and cytokine assay batch. This was based on prior work demonstrating significant confounding effects of each of these variables.^9^

Main effect of genetic risk: Linear regressions were used to assess the relationship between GRS for severe COVID‐19 and inflammatory marker levels. Log‐transformed pg mL^−1^ inflammatory marker levels represented our outcome variables, GRS our independent variable, with covariates as described above.

Genetic risk by age interaction: We multiplied age by our GRS to generate our interaction term. This was included in the regression described above, alongside the main effects of age and genetic risk.

Genetic risk by BMI interaction: We multiplied BMI by our GRS to generate our interaction term. This was included in the regression described above, alongside the main effects of BMI and genetic risk.

The false discovery rate (FDR) method was used to correct for the number of inflammatory markers in each set of analyses, whereby *P*_FDR_ < 0.1 was considered significant.

Histograms were used to confirm the normality of continuous variables, and standardised residuals (*z*‐scores) were generated for the purposes of scatterplots. Outliers were detected using the extreme studentised deviate test and excluded where they significantly deviated from the normal distribution. Graphs were generated using Prism 7 (GraphPad, San Diego, CA, USA); statistical analyses were performed using IBM SPSS Statistics version 26 (IBM, Armonk, NY, USA).

## Conflict of Interest

GB has received research funding from Eli Lilly. All other authors declare no relevant competing interests.

## Author Contributions

**Timothy Powell:** Conceptualization; Formal analysis; Investigation; Methodology; Supervision; Writing‐original draft; Writing‐review & editing. **Matthew** **Hotopf:** Funding acquisition; Resources; Writing‐review & editing. **Stephani L Hatch:** Funding acquisition; Resources; Writing‐review & editing. **Gerome Breen:** Funding acquisition; Investigation; Resources; Writing‐review & editing. **Rodrigo Roberto** **Rafagnin Duarte:** Conceptualization; Investigation; Supervision; Writing‐review & editing. **Douglas Nixon:** Funding acquisition; Investigation; Resources; Supervision; Writing‐review & editing.
